# Regulatory roles of RNA modifications in plant development and fruit ripening

**DOI:** 10.1007/s42994-025-00240-5

**Published:** 2025-08-08

**Authors:** Tianxiang Li, Junmei Huang, Guanqun Wang, Haoxuan Li, Peitao Lü

**Affiliations:** 1 National Key Laboratory of Tropical Crop Breeding, Institute of Tropical Bioscience and Biotechnology & Sanya Research Institute, Chinese Academy of Tropical Agricultural Sciences, Sanya, 572024 China; 2 College of Horticulture, Center for Plant Metabolomics, Haixia Institute of Science and Technology, Fujian Agriculture and Forestry University, Fuzhou, 350002 China; 3 School of Life Sciences and State Key Laboratory of Agrobiotechnology, The Chinese University of Hong Kong, Shatin, Hong Kong; 4 Department of Chemistry, Department of Biochemistry and Molecular Biology, and Institute for Biophysical Dynamics, The University of Chicago, Chicago, IL 60637 USA

**Keywords:** Epigenetics, Epitranscriptomics, Fruit maturation, Plant development

## Abstract

The emerging field of epitranscriptomics has revolutionized our understanding of post-transcriptional regulation in plant systems. This review focuses on cutting-edge discoveries in the area of RNA modification, with a particular emphasis on the N^6^-methyladenosine (m^6^A)-mediated regulatory networks that govern plant development and fruit maturation. We systematically summarize the spatiotemporal patterns of RNA modifications and their integration into phytohormone signaling cascades and responses to environmental stimuli. Advanced epitranscriptome sequencing platforms have identified evolutionarily conserved modification signatures across angiosperm species, while simultaneously revealing species-specific regulatory architectures. Despite substantial progress, our understanding of the molecular mechanisms that underlie RNA modifications, especially those other than m^6^A, remains limited. We propose an innovative roadmap that combines CRISPR-based writer/eraser manipulation, single-cell spatial epitranscriptomics, and synthetic biology approaches to harness RNA modification networks for precision agriculture. We also underscore the importance of interdisciplinary collaboration that integrates findings from biology, chemistry, physics, and computer science to decode the plant epitranscriptome. To enable precise control of postharvest physiology, future priorities should include the development of biosensors for specific modification types, the engineering of RNA modification–dependent translation control systems, and the development of RNA epigenetic editing tools.

## Introduction

The epitranscriptomic landscape of eukaryotic organisms includes over 170 chemically distinct RNA modifications, dynamically distributed across mRNA, tRNA, and rRNA molecules (Cantara et al. 2011; Boccaletto et al. 2018; Shen et al. 2019). These post-transcriptional modifications can be classified into two functional categories: (1) constitutive modifications that are essential for the structural integrity and basic functions of RNA, and (2) stimulus-responsive modifications that orchestrate RNA metabolism in a spatiotemporally precise manner. Emerging evidence shows that RNA chemical marks serve as molecular rheostats, integrating developmental cues, environmental signals, and epigenetic inputs to fine-tune gene expression networks (Shi et al. 2019; Yue et al. 2019). Recent mechanistic studies have revealed RNA modification–mediated regulatory pathways in plant stress adaptation. In *Arabidopsis thaliana*, the m^6^A reader protein evolutionarily conserved C-terminal region 8 (ECT8) functions as a salt-stress sensor by specifically binding to m^6^A-modified transcripts, triggering their decapping and degradation to maintain cellular homeostasis under saline conditions (Cai et al. 2024b). Similarly, the rice m^6^A eraser AlkB homolog 9 (ALKBH9) modulates male fertility through demethylation-mediated stabilization of *TAPETUM DEGENERATION RETARDATION* (*TDR*) and *GAMYB* mRNAs, which are critical for pollen development (Tang et al. 2024a). Despite these advances in understanding the roles of RNA modifications in vegetative growth and abiotic stress responses, much less is known about their specific functions in the development and maturation of horticultural fruit crops.

The epigenetic regulation of fruit maturation has been extensively characterized using multi-omics approaches. The fruitENCODE project systematically decoded chromatin state dynamics, revealing conserved regulatory circuits involving MADS-box and NAC transcription factors (TFs) that govern climacteric ripening (Lü et al. 2018; Li et al. 2025a). In tomato, a model system for fruit biology, DEMETER‐like DNA demethylase 2 (DML2) controls fruit ripening by erasing CG methylation at the *Ripening Inhibitor* (*RIN*) locus (Zhong et al. 2013; Liu et al. 2015; Lang et al. 2017), and DNA *Methyltransferase 1* (*MET1*)-mediated methylation patterns regulate parthenocarpy (Yang et al. 2019b). The histone modification machinery, including the H3K27 demethylase JmjC Domain-containing protein 6 (JMJ6) and histone deacetylases (SlHDA1/3, SlHDT1), further fine-tunes ripening kinetics through chromatin-based regulation of ethylene biosynthesis and signal transduction pathways (Guo et al. 2017a, b; Guo 2022).

Although these DNA- and histone-centric epigenetic mechanisms have been comprehensively reviewed, the RNA epitranscriptomic regulation of plant development remains largely uncharted. The present review bridges this critical knowledge gap by synthesizing recent breakthroughs in RNA modification biology, with a particular emphasis on the spatiotemporal regulation of fruit maturation cascades. We also discuss current technological limitations and propose an interdisciplinary roadmap to harness epitranscriptomic networks for precision horticulture.

## RNA modifications in plants

### Regulatory mechanisms of m^6^A modification

m^6^A modification, defined as methylation at the sixth nitrogen atom of adenine bases, is the most abundant and dynamically reversible post-transcriptional modification of eukaryotic mRNA and was first identified in mammals in 1974 (Desrosiers et al. 1974). The process of m^6^A modification involves three key regulatory components: methyltransferases (“writers”), which catalyze methylation, demethylases (“erasers”), which remove methyl groups, and recognition proteins (“readers”), which interpret the modification (Motorin and Helm 2022; Tan et al. 2024). In mammals, the METTL3–METTL14 (methyltransferase-like 3 and 14) heterodimer forms the catalytic core of the writer complex (Li et al. 1997; Liu et al. 2014; Ping et al. 2014; Wang et al. 2016a, b), assisted by auxiliary proteins such as Wilms tumor 1-associating protein (WTAP), Vir-like m^6^A methyltransferase associated (VIRMA), RNA-binding motif protein 15/15B (RBM15/15B), and Zinc finger CCCH domain-containing protein 13 (ZC3H13) (Ping et al. 2014; Patil et al. 2016; Wen et al. 2018; Yue et al. 2018). METTL16, a U6 adenosine methyltransferase, is also involved in m^6^A modification of human mRNA (Pendleton et al. 2017). The erasers fat mass and obesity-associated protein (FTO) and ALKBH5 are members of the ALKBH subfamily within the Fe(II)/2-oxoglutarate-dependent dioxygenase superfamily (Jia et al. 2011; Fu et al. 2013; Zheng et al. 2013; Xu et al. 2014; Zhang et al. 2016). Readers such as YTH domain-containing proteins (YTH) and RNA-binding proteins (RBPs) primarily recognize m^6^A-modified RNA and ensure the precise regulation of its stability, nuclear export, and translation (Wang et al. 2014, 2015; Alarcon et al. 2015; Meyer et al. 2015; Edupuganti et al. 2017).

**Fig. 1:**
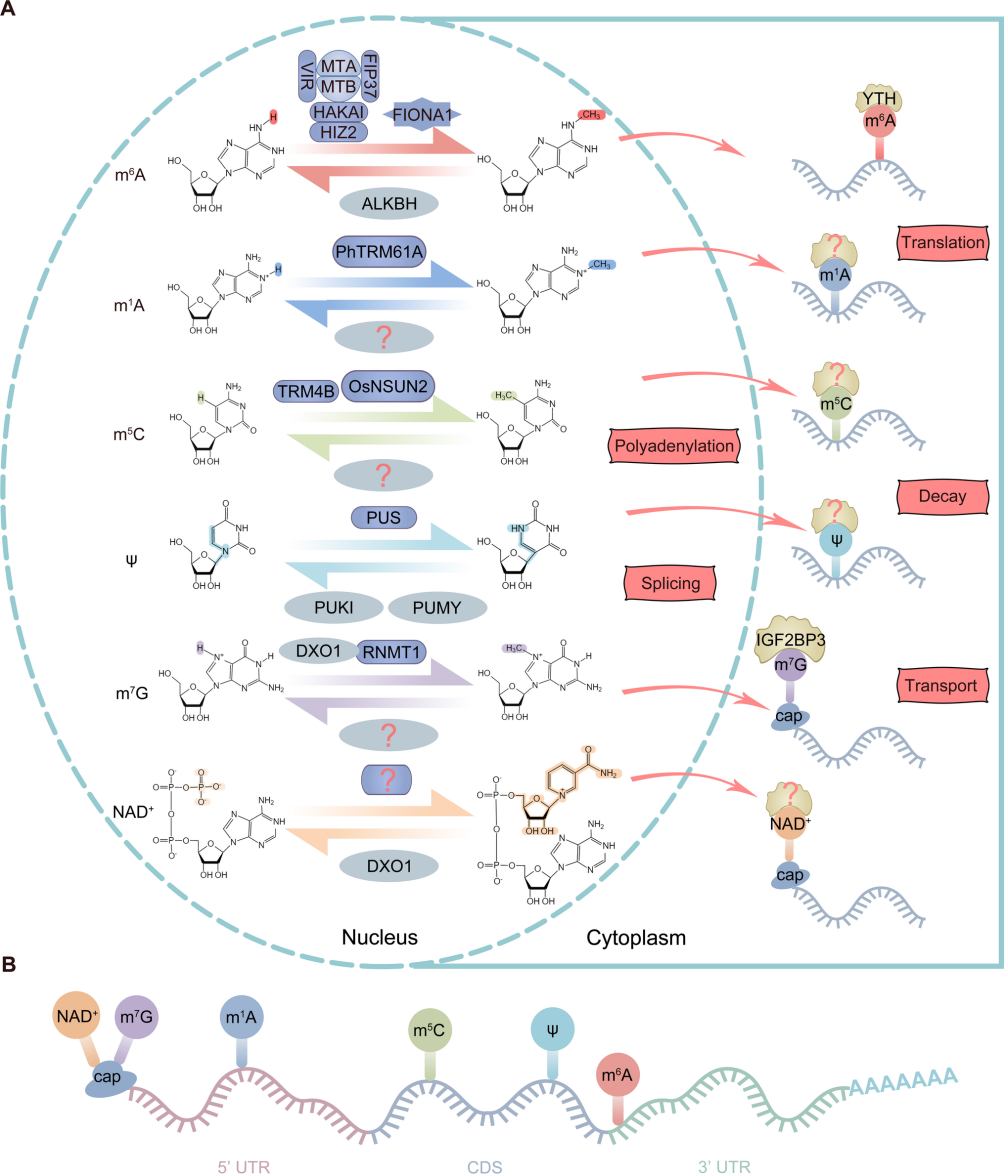
The regulatory mechanisms (**A**) and locations (**B**) of RNA modifications on mRNA. Different RNA modifications are enriched at different positions on mRNA and primarily influence gene expression by modulating mRNA splicing, polyadenylation, decay, transport, and translation. Parts of the figure were drawn using images from Biovisart (https://biovisart.com.cn)

In Arabidopsis, m^6^A deposition relies on interactions among methyltransferase A (MTA), methyltransferase B (MTB), FKBP12 interacting protein 37 kDa (FIP37), virilizer (VIR), the E3 ubiquitin-protein ligase Hakai (HAKAI), and HAKAI-interacting zinc finger protein 2 (HIZ2), which are homologs of mammalian METTL3, METTL14, WTAP, VIRMA, HAKAI, and ZC3H13, respectively (Fig. 1A) (Zhong et al. 2008; Shen et al. 2016; Ruzicka et al. 2017; Parker et al. 2021; Zhang et al. 2022a; Cai et al. 2024a). The MTA–MTB heterodimer serves as the catalytic core, with FIP37, VIR, and HAKAI as essential auxiliary subunits. The METTL16 homolog FIONA1, which functions as a monomer, was also identified as an m^6^A methyltransferase (Sun et al. 2022; Wang et al. 2022a; Xu et al. 2022). The dynamic regulation of m^6^A methylation in Arabidopsis relies on eraser enzymes, and the Arabidopsis genome encodes 14 members of the ALKBH family (Zhang et al. 2024a). Although no FTO orthologs are present, six ALKBH5 orthologs (AtALKBH9A, 9B, 9C, 10A, 10B, and 10C) have been identified in Arabidopsis (Liang et al. 2020), some of which have been shown to mediate m^6^A demethylation (Fig. 1A) (Duan et al. 2017; Liang et al. 2020; Martinez-Perez et al. 2021; Amara et al. 2022; Tang et al. 2022; Fan et al. 2023).

There are three types of m^6^A recognition proteins in mammals: YT512-B homology (YTH) proteins, heterogeneous nuclear ribonucleoproteins (HNRNPs), and some specific RBPs (Dominissini et al. 2012; Schwartz et al. 2013, 2014b; Wang et al. 2014; Alarcon et al. 2015; Meyer et al. 2015). In plants, all identified readers belong to the YTH family (Fig. 1A) (Arribas-Hernandez et al. 2018; Scutenaire et al. 2018; Wei et al. 2018; Hou et al. 2021; Song et al. 2021, 2023). Arabidopsis, rice, tomato, and wheat contain 13, 12, 9, and 13 YTH family members, respectively (Scutenaire et al. 2018; Arribas-Hernandez et al. 2020; Yin et al. 2021). The Arabidopsis YTH family comprises 11 YTHDF proteins (also termed Evolutionarily Conserved C Terminus or ECT proteins) and 2 YTHDC proteins (Scutenaire et al. 2018; Arribas-Hernandez et al. 2020). Cleavage and Polyadenylation Specificity Factor 30 (CPSF30) is one of the two YTHDC proteins. The *AtCPSF30* gene undergoes alternative polyadenylation (APA) to produce two transcripts, *AtCPSF30-S* and *AtCPSF30-L*, with the latter containing a YTH domain at its C terminus (Hou et al. 2021). Similar to those in mammals, m^6^A modifications in Arabidopsis are mainly enriched near the stop codon and 3′ UTR (Fig. 1B) and tend to occur at the consensus motif RRACH (R = A/G, H = A/C/U) (Luo et al. 2014; Shen et al. 2016).

Recent advances have enabled the detection of m^6^A modifications at a high level of resolution (Ge et al. 2023; Liu et al. 2023; Xiao et al. 2023b). The single-nucleotide resolution of m^6^A-selective allyl chemical labeling and sequencing (m^6^A-SAC-seq) has optimized motif discovery, revealing that m^6^A modification sites are primarily enriched in sequences containing RAC (R = A/G) and GAT motifs, the latter being a newly discovered plant-specific motif (Wang et al. 2024).

A number of advanced methods are used for transcriptome-wide profiling of m^6^A modifications: methylated RNA immunoprecipitation with next-generation sequencing (MeRIP-seq) enables transcriptome-wide surveys, albeit at modest resolution (Meyer et al. 2012); enzymatic strategies such as deamination adjacent to RNA modification targets sequencing (DART-seq) offer higher resolution and enable single-cell, transcriptome-scale mapping (Meyer 2019); chemical approaches like Glyoxal and nitrite-mediated deamination of unmethylated adenosine (GLORI) provide single-nucleotide resolution and enable absolute quantification (Shen et al. 2024); and Nanopore direct RNA sequencing (dRNA-seq) enables single-molecule identification of m^6^A modifications without the need for reverse transcription or amplification (Leger et al. 2021; Jain et al. 2022). In addition, machine learning–driven computational frameworks have been developed to predict potential m^6^A modification sites (Tsagkogeorga et al. 2022).

#### m^6^A modifications in plant development

**Fig. 2:**
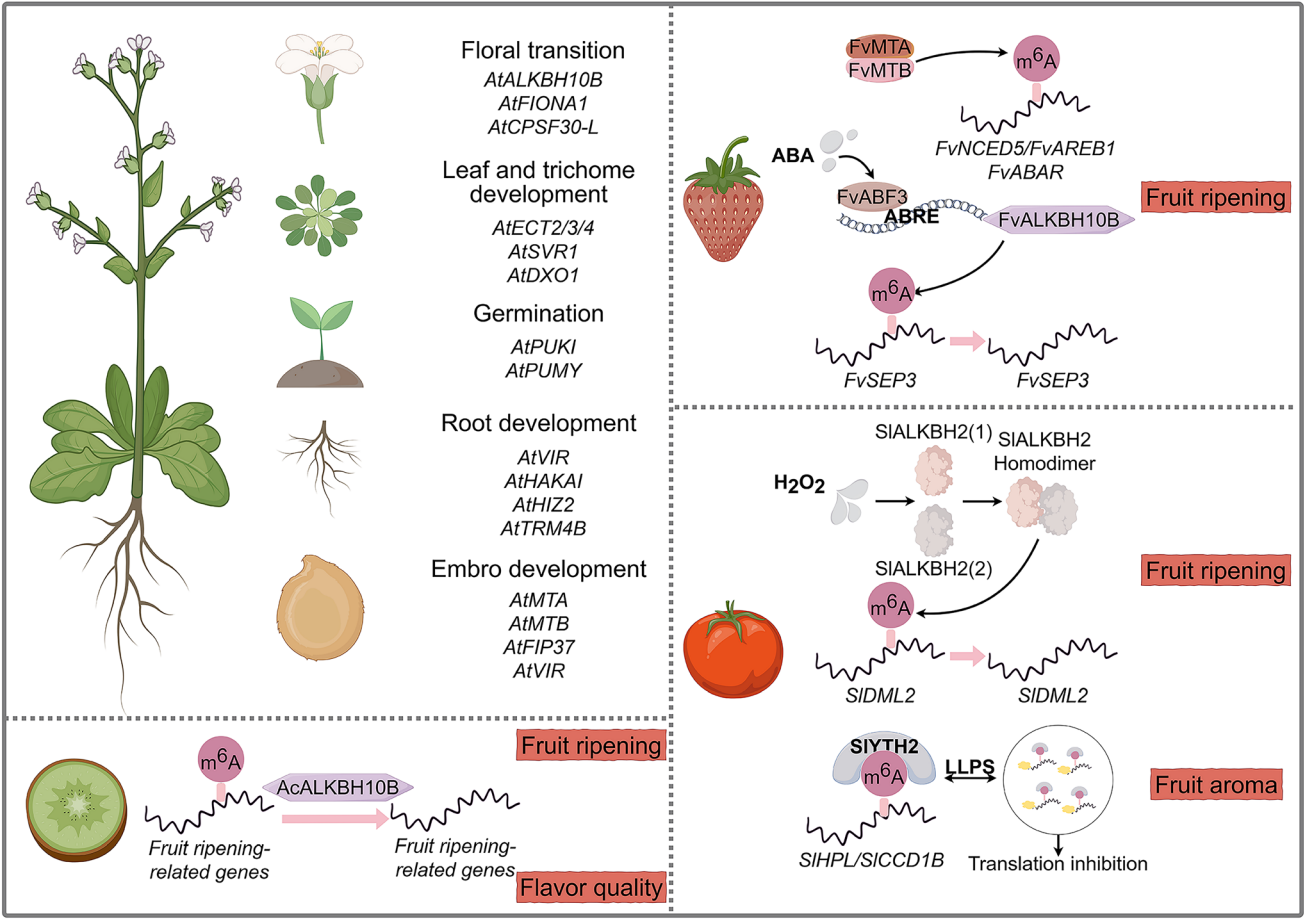
Biological functions of RNA modifications in plant development and fruit ripening. RNA modifications play a crucial role in the regulation of Arabidopsis development, including germination, the floral transition, and root, leaf, and trichome development. In horticultural plants, m^6^A modification plays a central role in fruit ripening and the modulation of attributes such as aroma and flavor through mRNA transcriptional regulation. The figure was designed using Figdraw (https://www.figdraw.com/#/)

m^6^A modification is crucial for plant development and fruit ripening (Fig. 2), playing key roles in processes such as sporogenesis, embryogenesis, organ morphogenesis, floral transition, and fruit ripening (Ruzicka et al. 2017; Arribas-Hernandez et al. 2020; Xu et al. 2022; Cheng et al. 2025; Zhou et al. 2025).

In Arabidopsis, the knockout or knockdown of the methylases *MTA*, *MTB*, *FIONA1*, *FIP37*, *VIRMA*, *HAKAI*, and *HIZ2* reduces overall m^6^A levels to varying degrees. Mutations in most writer genes (except for *HAKAI* and *FIONA1*) produce embryonic developmental defects (Shen et al. 2016; Ruzicka et al. 2017; Xu et al. 2022; Zhang et al. 2022a, 2022b). For example, FIP37-mediated m^6^A modification of shoot apical meristem regulators controls their mRNA stability, thereby determining shoot stem cell fate, and mutation of *FIP37* thus leads to overproliferation of the shoot apical meristem. In rice, FIP37 deficiency causes male sterility, as OsFIP37 facilitates the m^6^A methylation of threonine protease and NTPase mRNAs to regulate microsporogenesis and embryo development (Zhang et al. 2019a). OsFIP37 is also recruited by OsFAP1 to stabilize *OsYUCCA3* mRNA, enhancing the auxin synthesis necessary for male meiosis and pollen development (Cheng et al. 2022). A recent study showed that OsZAF activates *OsFIP37* expression, initiating a positive feedback loop (OsZAF–OsFIP37–OsYUCCA3–OsARF12) that modulates male meiosis in anthers (Cheng et al. 2025). Demethylase deficiencies can also influence plant sterility. For example, in rice, OsALKBH9 demethylates the m^6^A modification of *TDR* and *GAMYB* mRNAs; its knockout causes excessive accumulation of pollen exine and, ultimately, male sterility (Tang et al. 2024a). Likewise, the mutation of *ALKBH10B* in Arabidopsis also reduces fertility (Wang et al. 2022b).

m^6^A modification plays an important role in organ morphogenesis and the floral transition. For example, Arabidopsis ECT2 regulates 3′-UTR processing of precursor mRNAs (pre-mRNAs) in the nucleus and enhances mRNA stability in the cytoplasm, ultimately affecting trichome morphogenesis (Scutenaire et al. 2018; Wei et al. 2018). In addition, ECT2, ECT3, and ECT4 are functionally redundant and collectively regulate root, leaf, and flower morphogenesis (Scutenaire et al. 2018; Wei et al. 2018; Arribas-Hernandez et al. 2020). The floral transition sets the stage for flower morphogenesis, and m^6^A modification mediated by FIONA1 or ALKBH10B stabilizes the mRNAs of key flowering regulators, thereby controlling the floral transition (Duan et al. 2017; Sun et al. 2022; Xu et al. 2022; Cai et al. 2023). FIONA1 also regulates alternative splicing through m^6^A modification of U6 small nuclear RNA (snRNA), thereby affecting flowering time (Parker et al. 2022; Wang et al. 2022a). In addition, AtCPSF30-L can undergo phase separation and recognize far-upstream elements (FUEs), a type of polyadenylation signal, on mRNA to regulate alternative polyadenylation and affect plant flowering time (Song et al. 2021). In rice, YTH07 interacts with EARLY HEADING DATE6 (EHD6) to enhance the binding of m^6^A-modified RNA and trigger YTH07 relocation to RNP granules through phase separation, sequestering CONSTANS-like 4 (COL4) mRNA, reducing its protein abundance, and accelerating flowering (Cui et al. 2024).

m^6^A modification plays a critical role in plant light-signaling pathways. In Arabidopsis, blue light markedly enhances transcriptome-wide m^6^A methylation in a process orchestrated by the photoreceptor CRY2 (CRYPTOCHROME 2). Upon blue-light activation, CRY2 undergoes liquid–liquid phase separation (LLPS) to form photobodies that serve as molecular hubs for the recruitment of the m^6^A methyltransferase complex, thereby modulating m^6^A deposition. This alters the degradation rate of mRNAs that encode core circadian-clock components and fine-tunes the light responsiveness of the circadian system (Wang et al. 2021). A blue-light phenotypic screen revealed that *fio1* mutants show chlorophyll-deficiency traits similar to those of *cry1cry2*, suggesting that CRY-dependent, FIO1-specific m^6^A modification is crucial for chlorophyll homeostasis. Furthermore, blue-light-induced phase separation of the CRY2–SPA1–FIO1 complex enhances FIO1 methyltransferase activity, promoting the m^6^A modification and translation of target transcripts to support chlorophyll balance and blue-light adaptation (Jiang et al. 2023). The m^6^A modification thus mediates light responses by modulating transcript stability and translation through the light-activated recruitment of methyltransferase complexes by photoreceptors like CRY2.

m^6^A modification is also involved in the regulation of seed germination and seedling development by abscisic acid (ABA). In Arabidopsis, ABA treatment promotes ALKBH9B-mediated demethylation of *ABA INSENSITIVE 1* (*ABI1*) and *BRI1-EMS-SUPPRESSOR 1* (*BES1*), affecting their stability and ultimately regulating seed germination and early seedling development (Tang et al. 2022). In addition, ALKBH10B orchestrates developmental transitions through m^6^A-dependent mRNA stabilization and demethylates key floral integrators to modulate seed germination and seedling establishment by regulating ABA-responsive transcripts (Shoaib et al. 2021; Han et al. 2023).

#### m^6^A modification in plant stress adaptation

m^6^A is involved in plant responses to abiotic stresses, including salt, drought, and temperature extremes, as well as biotic stresses, such as viral infections. The expression of *AtMTA*, *AtMTB*, *AtFIP37*, and *AtVIR* is upregulated under salt stress, which increases the m^6^A modification of salt-stress response genes in Arabidopsis. Mutants such as pro*ABI3*::*MTA*/*mta*, *MTB*-RNAi, *fiona1*, *vir-1*, and *hakai* showed increased sensitivity to salt stress (Hu et al. 2021; Cai et al. 2024a). VIR- or FIONA1-mediated m^6^A methylation modulates reactive oxygen species (ROS) homeostasis by regulating the mRNA stability of salt-stress response factors (Hu et al. 2021; Cai et al. 2024a). In apple, MdMTA improves the stability and translation efficiency of stress-related mRNAs by enhancing their m^6^A modification, ultimately promoting lignin deposition and ROS scavenging under drought stress (Hou et al. 2022). Similarly, PtrMTA-mediated m^6^A methylation improves drought resistance in poplar through its effects on root and trichome development (Lu et al. 2020).

Demethylases also have important roles in stress responses. In Arabidopsis, ALKBHs have been implicated in the regulation of responses to salt, cold, heat, drought, and ABA. Each of the ALKBH proteins plays a role in at least one of these responses, and some contribute to all of them (Amara et al. 2022; Fan et al. 2023; Han et al. 2023), mainly by regulating the stability of stress-responsive transcripts through dynamic RNA methylation modifications. For example, AtALKBH9B is localized in cytoplasmic stress granules (SGs) under heat stress; there, it facilitates the demethylation of *Onsen* retrotransposon RNA, promoting the release of *Onsen* RNA from SGs and enabling its transposition in response to heat stress (Fan et al. 2023). Furthermore, GhALKBH10 enhances plant tolerance to salt and drought stress by demethylating stress-related transcripts (Cui et al. 2022).

Roles of m^6^A modification in biotic stress responses have also been reported. Overexpression of tobacco *NbMETTL1* and *NbMETTL2*, homologs of *Arabidopsis FIONA1*, reduced *plum pox virus* (PPV) accumulation in tobacco, indicating that NbMETTL1/2 are involved in its antiviral response (Yue et al. 2023). A genome-wide association study in wheat revealed that TaMTB is a positive regulator of *Wheat Yellow Mosaic Virus* (WYMV) infection. TaMTB relocates to cytoplasmic viral replication centers by interacting with the Nib replicase of WYMV, stabilizing viral RNA and promoting viral infection. Moreover, a SNP in *TaMTB* (SNP176A/C) is associated with WYMV susceptibility, and wheat varieties carrying *TaMTB176C* exhibit greater susceptibility (Zhang et al. 2022c). ALKBH9B has been shown to remove m^6^A modifications from *alfalfa mosaic virus* RNA, playing a role in virus infection of Arabidopsis (Martinez-Perez et al. 2017, 2021). In addition, ClALKBH4B may be involved in the early response of watermelon to *Cucumber green mottle mosaic virus* (He et al. 2021). Arabidopsis ECT1 functions in plant stress responses mediated by salicylic acid (SA): SA or pathogen induction prompts ECT1 to undergo LLPS and bind to m^6^A-modified mRNA, forming cytoplasmic biomolecular condensates. The degradation or storage of modified mRNAs in cytoplasmic condensates slows the translation of SA-response genes, limiting the SA-induced stress response (Lee et al. 2024).

### Non-m^6^A modifications

#### m^1^A modification

m^1^A modification contributes to the structural stability, post-transcriptional regulation, and translation efficiency of RNA and is prevalent in tRNA, rRNA, and mRNA across bacteria, animals, and plants (Dominissini et al. 2016). The primary m^1^A modification sites on tRNA are positions 9, 14, 22, 57, and 58. m^1^A modification at position 58 (m^1^A58) is catalyzed by the TRM6/TRM61 methyltransferase complex (Oerum et al. 2017), in which TRM61 contains a conserved motif for binding the methyl donor S-adenosylmethionine (SAM) and TRM6 is crucial for tRNA binding. The m^1^A modification of tRNA mediated by TRM61/TRM6 is crucial for Arabidopsis embryonic development (Tang et al. 2020a; Aslam et al. 2022). Knockdown of *AtTRM61* or knockout of *AtTRM6* in Arabidopsis led to arrested embryonic development and seed abortion, whereas the conditionally complemented line *Attrm61/LEC1pro::AtTRM61* showed normal seed development (Tang et al. 2020a). Another study suggested that AtTRM61/AtTRM6 are essential for both embryo and endosperm development and may be regulated by the MEKK1–MKK1/2–MPK4 signaling cascade (Aslam et al. 2022).

Transcriptome-wide m^1^A mapping by m^1^A-seq (Dominissini et al. 2016) or m^1^A-ID-seq (Li et al. 2016) can now reveal the landscape of m^1^A modifications on mRNA with single-base resolution (Li et al. 2017). In mammals, m^1^A modifications are predominantly enriched near the start codon and within the 5′ UTR, and those in the 5′ UTR and mRNA cap are closely linked to translation efficiency (Dominissini et al. 2016; Li et al. 2017). Like m^6^A modifications, m^1^A modifications of mRNA can be reversed by the demethylase ALKBH3 (Li et al. 2016; Zhang et al. 2024b). m^1^A modifications of mRNA are also enriched in the 5′ UTR in *Petunia hybrida*, (Fig. 1B), and dot blotting and LC–MS/MS showed that m^1^A modifications were more abundant in mRNA than in rRNA. mRNA m^1^A levels varied across roots, stems, leaves, and corollas of *P. hybrida*, and ethylene treatment of the corolla reduced the m^1^A modification levels of its mRNA. In addition, silencing of PhTRMT61A caused abnormal leaf development. Together, these results suggest that plants can modulate the m^1^A levels of mRNA to regulate development and respond to environmental cues (Yang et al. 2020).

#### m^5^C modification

m^5^C modification is widespread in various RNAs and is regulated by writers, erasers, and readers. In animals, m^5^C writers consist primarily of NOL1/NOP2/Sun (NSUN) family proteins and DNA methyltransferase (DNMT) family proteins, which contain a Rossman-fold catalytic domain and SAM binding sites (Xue et al. 2020). m^5^C erasers are mainly Ten-Eleven Translocation (TET) proteins, which convert RNA 5-methylcytosine (5mC) to 5-hydroxymethylcytosine (5hmC), 5-formylcytidine (5fC), and 5-carboxycytidine (5caC) (Xue et al. 2020). m^5^C readers include RNA binding protein Aly/REF export factor (ALYREF) (Yang et al. 2017) and Y-box binding protein 1 (YB1) (Yang et al. 2019c); they play crucial roles in precise regulation by recognizing m^5^C-modified RNA. m^5^C modifications of tRNA and rRNA have been identified across a broad spectrum of photosynthetic organisms, from the single-celled alga *Nannochloropsis oculata* to the angiosperm *Arabidopsis*, and their modification sites are highly conserved. In Arabidopsis, nuclear tRNA methylation requires two conserved methyltransferases: transfer RNA aspartic acid methyltransferase 1 (TRDMT1) and tRNA-specific methyltransferase 4B (TRM4B), which are homologous to human DNMT2 and NSUN2. These two enzymes play a crucial role in regulating translation (Burgess et al. 2015). m^5^C modification of mRNA has also been widely reported in various plants and in different tissues and developmental stages of *Arabidopsis*. Notably, in seedlings, the m^5^C/C ratio is significantly higher in mRNA than in other RNA types (Cui et al. 2017). In addition, m^5^C is found mainly in the CDSs of *Arabidopsis* genes (Fig. 1B), and two major m^5^C peaks located after the start codon and before the stop codon are associated with mRNA translation efficiency (Cui et al. 2017).

The m^5^C modification of mRNA is fundamental to plant development and environmental responses. TRM4B-mediated m^5^C modification of the root development factors *SHY2* and *IAA16* regulates root morphogenesis in Arabidopsis, and mutation of *TRM4B* causes root developmental defects (Cui et al. 2017; David et al. 2017). The long-distance transport of mRNA is regulated by m^5^C modification. *Translationally Controlled Tumor Protein 1* (*TCTP1*) has been identified as a mobile transcript that can cross graft junctions to distant tissues. The reduced level of m^5^C modification in the *dnmt2 (trdmt1) nsun2b (trm4b)* double mutant renders *TCTP1* mRNA immobile, preventing its movement through the phloem to the root cells, where it would normally promote root growth and development (Yang et al. 2019a). m^5^C modification also plays an important role in the high-temperature adaptation of rice. The rice *nsun2* mutant exhibits a heat-hypersensitive phenotype due to reduced m^5^C modification of chloroplast-development genes, leading to decreased translation efficiency. This results in disrupted chloroplast function and the accumulation of light-dependent ROS (Tang et al. 2020b).

#### ac^4^C acetylation modifications

N^4^-acetylcytidine (ac^4^C) is the only known acetylation-type RNA modification; it occurs at the N^4^ position of cytidine and has been identified in tRNA, rRNA, and mRNA across mammals (Arango et al. 2018), archaea (Sas-Chen et al. 2020), and plants (Wang et al. 2023). In mammals, ac^4^C is installed by the sole known writer, N-acetyltransferase 10 (NAT10), which has both acetyltransferase and RNA-binding domains. Recently, the NAD-dependent protein deacetylase Sirtuin-7 (SIRT7) was identified as an rRNA deacetylase in mammals (Xu et al. 2021), suggesting that RNA acetylation is also dynamically reversible. However, such regulatory mechanisms remain unexplored in plants.

ac^4^C modification is widespread in plants and plays important roles in growth and development, stress responses, and fruit ripening. In *Arabidopsis*, rice, and tomato, ac^4^C modifications are predominantly enriched in the CDS regions of mRNA. Two homologs of NAT10 have been identified in Arabidopsis and named N-ACETYLTRANSFERASEs FOR CYTIDINE IN RNA (ACYRs). The AtACYRs are essential for gametophytic transmission and vegetative growth. Single mutants of *ACYR1* or *ACYR2* exhibit reduced ac^4^C levels but remain viable, whereas mutation of both genes causes embryo mortality (Wang et al. 2023). Rice encodes the single homolog *OsNAT10*/*OsACYR*, whose disruption causes a global reduction in ac^4^C modification levels, particularly of photosynthesis-related genes, resulting in reduced photosynthetic capacity and developmental defects (Cai et al. 2025). The ac^4^C modification has also been shown to regulate rice resistance to *Magnaporthe oryzae*. Upon pathogen infection, upregulation of *OsNAT10*/*OsACYR* enhances the translation of defense-related genes and promotes immune activation (Lu et al. 2024). In tomato, dynamic changes in ac^4^C levels have been observed during fruit ripening: ac^4^C levels decrease in the fruit of wild-type Ailsa Craig (AC) but increase in those of the ethylene-insensitive *never ripe* (*nr*) mutant, suggesting that ac^4^C is involved in the regulatory network that governs tomato fruit ripening (Ma et al. 2024a).

#### Ψ modification

Pseudouridine (Ψ) is an isomerized product of uridine (U). Ψ modifications regulate various aspects of RNA function, including rRNA precursor processing, ribosome assembly, and protein translation (Ofengand 2002); modulation of tRNA structure to enhance decoding accuracy and protein-synthesis fidelity (Davis 1995); snRNA assembly and pre-mRNA splicing (Zhao and Yu 2004; Karijolich and Yu 2010); and mRNA translation and protein synthesis (Kariko et al. 2008; Schwartz et al. 2014a, b). Ψ modifications are mediated by two mechanisms: one relies on RNA-guided A/ACA ribonucleoproteins for catalysis, and the other is catalyzed by pseudouridine synthases (PUSs) (Charette and Gray 2000; Ofengand 2002). *Arabidopsis*, maize, rice, soybean, and tomato contain 20, 22, 22, 31, and 19 PUS family members, respectively (Xie et al. 2022). In *Arabidopsis* mRNA, Ψ modifications are distributed across the 5′ UTR, CDS, and 3′ UTR (Fig. 1B) and are predominantly enriched at the first codon position, with UUC being the most frequently pseudouridylated site. Ψ modifications have also been detected in non-coding RNAs, including tRNA, mRNA, snRNA, and snoRNA (Sun et al. 2019).

Ψ modification plays an important role in plant development and environmental responses. SUPPRESSOR OF VARIEGATION 1 (SVR1) is a chloroplast-localized PUS in Arabidopsis. Mutation of *SVR1* eliminates certain Ψ modifications in chloroplast 23S, 16S, 5S, and 4.5S rRNA, leading to a reduction in chloroplast translation. As a result, the plant exhibits a yellow-green color and a significantly reduced size (Yu et al. 2008). *SVR1* mutation also results in an insensitivity to phosphate starvation, likely due to impaired chloroplast translation (Lu et al. 2017). In rice, *OsTCD3*, also referred to as *OsPUS1*, encodes a chloroplast-localized pseudouridine synthase that is crucial for chloroplast development, particularly under low-temperature conditions (Lin et al. 2020; Wang et al. 2022c). Loss-of-function mutants (*tcd3*/*ospus1*) exhibit chlorotic leaves and eventual mortality at low temperatures, primarily due to disrupted chloroplast rRNA processing, impaired tRNA metabolism, and compromised ribosome biogenesis and translation. In addition, *OsPUS1*/*TCD3* is induced by low temperatures and maintains redox homeostasis by preventing ROS overaccumulation, highlighting the essential role of pseudouridine modification in rice cold-stress responses and early leaf development. PUSs localized in the mitochondria are also crucial for plant growth and development. Arabidopsis *FCS1* (*Leaf Curly and Small 1*) encodes a mitochondrial PUS, and the *fcs1* mutant displays delayed development and reduced fertility. Loss of *FCS1* significantly reduces the pseudouridylation of mitochondrial 26S rRNA, leading to the disruption of mitochondrial protein translation. FCS1-mediated pseudouridine modification of mitochondrial 26S rRNA is therefore essential for maintaining mitochondrial function and plant development (Niu et al. 2022). BID-seq technology has recently been used to generate Ψ modification maps at single-nucleotide resolution in four plant species (Arabidopsis, rice, maize, and soybean), covering tRNA, rRNA, and mRNA. These analyses revealed that Ψ modifications regulate translation in a site- and context-dependent manner. Specifically, Ψ in rRNA differentially influences mRNA translation depending on the 5′ UTR and CDS lengths, whereas Ψ in tRNA loop regions enhances codon-specific translation efficiency. Ψ sites in mRNA exhibit strong tissue specificity and are associated with increased translational efficiency and reduced mRNA stability, highlighting the multilayered regulatory role of Ψ in plant gene expression (Li et al. 2025b).

The metabolism of Ψ is also crucial for plant growth and development. In *Arabidopsis*, Ψ is primarily metabolized in peroxisomes. First, the pseudouridine kinase PUKI phosphorylates Ψ to form 5′-pseudouridine monophosphate (5′-ΨMP), which is subsequently hydrolyzed into uridine and ribose-5-phosphate by the ΨMP glycosylase PUMY. Moreover, excessive accumulation of Ψ and ΨMP leads to delayed seed germination in mutants. To avoid the cytotoxic effects of cytoplasmic Ψ accumulation, PUKI and PUMY enzymes are localized in the peroxisomes, ensuring efficient Ψ metabolism (Chen and Witte 2020).

#### m^7^G modification

The N^7^-methylguanosine (m^7^G) modification is widely present in eukaryotic RNA. In tRNA, the m^7^G modification occurs primarily at position 46 (Alexandrov et al. 2002; Li et al. 2023a) and is crucial for stabilizing the tRNA tertiary structure and influencing translation efficiency. In 18S rRNA, m^7^G occurs mainly at position 1575 in yeast (Figaro et al. 2012) and at position 1639 in humans (Zorbas et al. 2015), contributing to rRNA structure and stability. In mRNA, m^7^G is added to the 5′ cap by RNA guanosine-7 methyltransferase (RNMT) after transcription initiation, thereby regulating mRNA stability, splicing, translation efficiency, and transport. m^7^G modifications are also enriched in the internal regions of mRNA (Zhang et al. 2021). The tRNA methyltransferase METTL1–WDR4 complex acts as the m^7^G writer for mRNA, and knockdown of METTL1 reduces the translation efficiency of certain transcripts (Zhang et al. 2019c). Recent studies have identified potential m^7^G readers, such as the IGF2BP family (IGF2BP1–3), among which IGF2BP3 has been shown to regulate mRNA degradation by binding to m^7^G on mRNA (Fig. 1A) (Liu et al. 2024a).

Plant development and environmental responses require regulation through m^7^G modification. In Arabidopsis, homozygous mutation of the m^7^G methyltransferase RNMT is lethal, whereas the abnormal embryos of heterozygotes are arrested at the globular stage (Xiao et al. 2023a). In addition, the well-established NAD-decapping enzyme DXO1 plays an important role in m^7^G modification, activating RNMT1 to promote methylation of the guanosine cap to form the m^7^G cap (Xiao et al. 2023a). Recently, Arabidopsis RID2 was identified as a methyltransferase responsible for m^7^G modification (Liu et al. 2024b). RID2 interacts with MYB3R-like to activate WUS transcription, methylating the 5′ cap of WUS mRNA to protect it from degradation. Under heat stress, MYB3R-like/RID2 is suppressed, leading to the decapping of WUS mRNA and reduced WUS transcript levels. This releases the repression of WUS on protein folding in stem cells, enhancing stem cell thermotolerance by reducing the accumulation of misfolded proteins. In rice, Cd treatment significantly reduces m^7^G levels in both the 5′ cap and internal regions of mRNA, a process potentially mediated by the m^7^G decapping genes *OsDCPL1/2/3* (Chu et al. 2018).

#### NAD^+^ capping modification

The non-canonical nicotinamide adenine dinucleotide (NAD^+^) cap is found on the RNA of both eukaryotic and prokaryotic organisms (Chen et al. 2009; Cahova et al. 2015; Jiao et al. 2017; Walters et al. 2017; Zhang et al. 2019b). RNA polymerase (RNAP) uses NAD^+^ instead of ATP as the initiating nucleotide, thereby adding NAD^+^ to the 5′ end of the RNA during de novo transcription initiation (Bird et al. 2016). NAD-RNA decapping affects RNA decay and stability. In mammals, NAD-RNA undergoes deNADding by DXO, resulting in 5′ monophosphorylated RNA, which is further degraded by DXO through its 5′–3′ exoribonuclease activity (Jiao et al. 2017). Likewise, Arabidopsis contains a single DXO with deNADding and 5′–3′ exonuclease activity, and a mutation in *DXO1* causes growth retardation and developmental defects in lateral roots, leaves, pistils, and siliques (Kwasnik et al. 2019; Pan et al. 2020).

In Arabidopsis, NAD^+^ modifications are primarily enriched in protein-coding genes. Gene Ontology (GO) enrichment analysis showed that these genes are involved in processes such as photosynthesis, translation, and responses to cytokinin and stress (Zhang et al. 2019b). Loss of *DXO1* function causes developmental defects in Arabidopsis, with the *dxo1* mutant showing a general downregulation of genes involved in photosynthesis and an upregulation of defense-related genes (Pan et al. 2020). However, rescuing the developmental defects of the mutant does not require enzymatically active DXO1 (Pan et al. 2020). Furthermore, DXO1-mediated 5′ NAD^+^ decapping is essential for the normal response of *Arabidopsis* seeds to ABA, and ABA treatment leads to dynamic changes in NAD^+^ modification of plant mRNA (Yu et al. 2021). DXO1 also plays a capping role in m^7^G modification in *Arabidopsis* and has been identified as an activator of the m^7^G methyltransferase RNMT1 (Xiao et al. 2023a). Thus, DXO fine-tunes the dynamic balance between alternative RNA cap structures (Yang and Cao 2023).

### RNA modifications regulate fruit development and ripening

Fruit development and ripening are critical processes in the life cycle of horticultural plants and involve the coordinated regulation of multiple molecular signals. Hormones, TFs, epigenetic mechanisms, and environmental factors coordinate to regulate fruit traits, including morphology, storage potential, disease resistance, sweetness, and aroma. As forms of post-transcriptional regulation, RNA modifications participate in the regulation of fruit development and maturation by influencing RNA stability, splicing, transport, and translation (Fig. 2) (Zhou et al. 2019b, 2021; Hu et al. 2022; Yin et al. 2022; Bian et al. 2024; Su et al. 2024).

RNA modification regulates tomato fruit development and ripening (Zhang et al. 2024c). In tomato, the levels of both DNA m^5^C modification and mRNA m^6^A modification gradually decrease during fruit ripening. In addition, the overall levels of both modifications are upregulated in the ripening-deficient epigenetic mutant *Colorless non-ripening* (*Cnr*), indicating a similar dynamic pattern of DNA methylation and mRNA methylation. The m^6^A demethylase SlALKBH2 demethylates and stabilizes the mRNA of the DNA demethylase gene *SlDML2*, thereby accelerating fruit ripening, and this finding establishes a molecular link between DNA methylation and mRNA m^6^A methylation (Zhou et al. 2019b). The SlALKBH2 protein can also be oxidized by H_2_O_2_ and form homodimers through intermolecular disulfide bonds; this significantly enhances its structural stability, thereby positively regulating fruit ripening by demethylation of *SlDML2*. By contrast, the NADPH-dependent thioredoxin reductase C (NTRC) catalyzes the reduction of SlALKBH2, ultimately delaying fruit ripening (Zhou et al. 2025). This mechanism reveals the intrinsic connection between reactive oxygen signaling and the epigenetic regulation of fruit ripening. In addition, during fruit development and expansion, overall m^6^A modification levels increase and are positively correlated with mRNA abundance. The methyltransferase inhibitor 3-deazaneplanocin A and the demethylase inhibitor meclofenamic acid both prevented the expansion of tomato fruit, but the former accelerated fruit ripening whereas the latter delayed it, highlighting the complex mechanisms by which m^6^A modification regulates tomato fruit development and ripening (Hu et al. 2022). A total of 24 m^6^A-related genes have been identified in tomato (Shen et al. 2022; Yin et al. 2021), including *SlYTH1* and *SlYTH2*, whose knockdown leads to dwarfism and delays fruit ripening (Yin et al. 2022; Ao et al. 2023). In addition, knockout of *SlYTH2* induces fruit aroma production. SlYTH2 binds to the mRNAs of the aroma synthesis genes *hydroperoxide lyase* (*HPL*) and *Carotenoid cleavage dioxygenase 1B* (*CCD1B*) in an m^6^A-dependent manner and interacts with translation-related factors such as translation initiation factors, forming protein–RNA condensates through LLPS, inhibiting mRNA translation and protein accumulation, and thus regulating the aroma quality of the fruit (Bian et al. 2024).

Other types of RNA modification also play critical roles in tomato fruit ripening (Guo et al. 2022a; Ma et al. 2024a, b). Analysis of global m^1^A, m^5^C, and ac^4^C modification levels in wild-type AC and the ethylene-insensitive *nr* mutant revealed that m^1^A and ac^4^C levels progressively decrease during fruit maturation (Ma et al. 2024a, b), whereas m^5^C levels increase (Guo et al. 2022a). These findings suggest that distinct RNA modifications may exert different regulatory effects during tomato fruit ripening. Because *nr* and AC are genetically distinct, well-characterized genotypes for the study of climacteric fruit ripening, the differences between them further highlight the regulatory potential of RNA modifications in this process. For example, *nr* fruits exhibit lower m^1^A levels than AC fruits (Ma et al. 2024b) and ac^4^C levels decrease in wild-type AC fruits but increase in the *nr* mutant during fruit ripening (Ma et al. 2024a).

Strawberry fruit ripening is also regulated by m^6^A modifications (Zhou et al. 2021; Tang et al. 2024b). Overall, m^6^A modifications are primarily enriched at the stop codon and 3′-UTR regions of strawberry fruit mRNA. However, as the fruit ripens, m^6^A modifications increase in the CDS region near the start codon and decrease in the 3′ UTR. This dynamic redistribution suggests a regulatory role for m^6^A modifications during fruit ripening. Genes with m^6^A-enriched CDS regions are generally upregulated during fruit ripening, whereas m^6^A enrichment in the 3′-UTR region is negatively correlated with expression levels. The list of genes with m^6^A-enriched CDS regions includes key genes in the ABA signaling pathway that are essential for strawberry ripening. Moreover, the methyltransferases MTA and MTB facilitate strawberry ripening by enhancing the mRNA stability of *NCED5* and *AREB1* and promoting the translation of *ABAR* through the m^6^A modification pathway. Overall, m^6^A regulates the ripening of non-climacteric strawberry fruits by increasing the transcript abundance or translation efficiency of ABA pathway genes, a process that is different from that reported in climacteric tomato fruit (Zhou et al. 2021). The demethylase ALKBH10B also positively regulates strawberry fruit ripening, but its mechanism is distinct from that of MTA and MTB. ABA signaling induces *ALKBH10B* expression via the ABF3 TF, leading to increased ALKBH10B levels. ALKBH10B removes m^6^A modifications from the 3′ UTR of *SEP3*, thereby stabilizing its mRNA. SEP3 then promotes the expression of ripening-related genes, ultimately facilitating fruit ripening. Thus, for ripening-associated genes, maintaining appropriate m^6^A levels during fruit ripening is essential. Disruption of either m^6^A methyltransferases or demethylases may disturb the homeostasis of these genes, thereby affecting fruit ripening (Tang et al. 2024b).

The ripening of kiwifruit is also regulated by the m^6^A demethylase ALKBH (Su et al. 2024), with m^6^A modifications primarily enriched in the mRNA stop codon and 3′-UTR regions. During fruit ripening, m^6^A modification levels gradually decrease and exhibit a clear negative regulatory relationship with mRNA levels during maturation, indicating that m^6^A modifications play an inhibitory role in kiwifruit ripening. Additional research indicates that the demethylase AcALKBH10 mainly regulates m^6^A levels in genes associated with ripening, thereby controlling the accumulation of soluble sugars and organic acids during the ripening process and ultimately affecting fruit ripening and quality formation.

In addition to directly regulating fruit development and ripening, RNA modifications also influence other aspects of development and disease resistance in horticultural plants, including systemic transport in *Cucurbitaceae* (Li et al. 2023b), low nitrogen and powdery mildew tolerance in apple (Guo et al. 2022a, b; 2023), leaf development and heterosis in tea plants (Liao et al. 2025), fire blight resistance in pear (Han et al. 2021), and petal and leaf development in petunia (Yang et al. 2020). Biotechnological applications based on epigenetic research offer new approaches for studying the growth and development of horticultural plants, particularly fruit development and ripening. These approaches have the potential to not only improve fruit quality but also extend shelf life and improve transportability.

## Future perspectives

Detection of RNA modifications in plants currently relies primarily on antibody-based affinity enrichment methods. Although conventional MeRIP-seq cannot measure modification rates at single-nucleotide resolution in mammalian RNA, techniques such as BID-seq (Dai et al. 2023), miCLIP-seq (George et al. 2017), m^1^A-quant-seq (Zhou et al. 2019a), PRAISE-seq (Zhang et al. 2023), and GLORI (Shen et al. 2024) have demonstrated single-base resolution capabilities. Nonetheless, they have not yet been applied to plants. The m^6^A-SAC-Seq technique, which offers single-nucleotide resolution, has been successfully applied to Arabidopsis and rice (Ge et al. 2023; Wang et al. 2024), significantly advancing our understanding of m^6^A-mediated regulation in plant development. Future investigations into the roles of RNA modifications in plant growth and development should therefore prioritize the development and application of ultra-high-resolution detection technologies.

In plants, recent research has focused predominantly on m^6^A modifications, and our understanding of other RNA modifications, such as m^5^C, m^1^A, Ψ, and m^7^G, remains limited. Studies on RNA modifications during fruit development and maturation have been centered almost exclusively on m^6^A. In addition, many RNA modification enzymes, including writers, erasers, and readers, remain unidentified in plants. Gene-editing techniques that specifically alter the RNA modification level of a single target gene are needed to precisely modify RNA modification sites. CRISPR/dCas13-based RNA methylation and demethylation editing tools have recently been applied to plants, successfully depositing and removing m^6^A modifications on specific RNA transcripts (Shi et al. 2024). Future advances and widespread applications of this technology in plants are essential for agricultural development.

Synthetic biology involves modular design, programmability, and interdisciplinary integration to enable the predictable, reproducible, and application-oriented optimization of biological systems. Currently, the creation of modified RNA relies on solid-phase synthesis combined with enzymatic ligation. In theory, solid-phase synthesis enables modifications at any nucleotide of an RNA molecule; however, the length of the synthesized RNA is limited to approximately 100 nucleotides (Flemmich et al. 2024). With technological advances, future research should focus on developing methods that enable modifications at any desired RNA position while overcoming length limitations.

Recently, single-cell transcriptomics and spatial transcriptomics have become widely used in plant research. Single-cell sequencing provides genetic information at the cellular level and reveals intra-population heterogeneity (Seyfferth et al. 2021), whereas spatial transcriptomics enables real-time monitoring of dynamic changes (Lv et al. 2024). Future studies should integrate single-cell transcriptomics, spatial transcriptomics, and epitranscriptomics to systematically investigate how RNA modifications influence gene expression and signaling pathways. Ultimately, combining these approaches with synthetic biology and CRISPR/dCas13 will pave the way for precise modification of agricultural crops.

The study of RNA modifications is transitioning from descriptive phenomenology to mechanistic dissection and applied innovation. Moving forward, it will be essential to establish plant-specific RNA modification databases, develop precise RNA-editing tools, and decode the dynamic interactions between RNA modifications and environmental factors. By harnessing interdisciplinary advances, RNA modification research holds the potential to serve as a transformative engine for intelligent crop design, offering groundbreaking solutions for sustainable agriculture.

## Data Availability

Data sharing is not applicable for this manuscript, as no datasets were generated or analyzed during the present study.
